# Traffic Signal Synchronization in the Saturated High-Density Grid Road Network

**DOI:** 10.1155/2015/532960

**Published:** 2015-01-13

**Authors:** Xiaojian Hu, Jian Lu, Wei Wang, Ye Zhirui

**Affiliations:** ^1^Jiangsu Key Laboratory of Urban ITS, Southeast University, Sipailou No. 2, Nanjing 210096, China; ^2^Jiangsu Province Collaborative Innovation Center of Modern Urban Traffic Technologies, Sipailou No. 2, Nanjing 210096, China; ^3^Beijing Key Laboratory for Cooperative Vehicle Infrastructure Systems and Safety Control, Sipailou No. 2, Nanjing 210096, China

## Abstract

Most existing traffic signal synchronization strategies do not perform well in the saturated high-density grid road network (HGRN). Traffic congestion often occurs in the saturated HGRN, and the mobility of the network is difficult to restore. In order to alleviate traffic congestion and to improve traffic efficiency in the network, the study proposes a regional traffic signal synchronization strategy, named the long green and long red (LGLR) traffic signal synchronization strategy. The essence of the strategy is to control the formation and dissipation of queues and to maximize the efficiency of traffic flows at signalized intersections in the saturated HGRN. With this strategy, the same signal control timing plan is used at all signalized intersections in the HGRN, and the straight phase of the control timing plan has a long green time and a long red time. Therefore, continuous traffic flows can be maintained when vehicles travel, and traffic congestion can be alleviated when vehicles stop. Using the strategy, the LGLR traffic signal synchronization model is developed, with the objective of minimizing the number of stops. Finally, the simulation is executed to analyze the performance of the model by comparing it to other models, and the superiority of the LGLR model is evident in terms of delay, number of stops, queue length, and overall performance in the saturated HGRN.

## 1. Introduction

Since the 1990s, the New Urbanism Movement has inspired several urban road network development trends, including increased use of the high-density grid road network (HGRN). The structure of the HGRN is the orthogonal checkerboard pattern, with narrow two-lane or four-lane roads, which are spaced approximately 100 to 300 meters apart. The density of roads in the HGRN is uniform, and there is no significant difference in the road grade.

The primary characteristic of the HGRN is homogeneity. In a district of HGRN, the distribution of the population in each block is uniform and the change in the intensity of the land use is small, so the amount of traffic volume generated in each unit area is almost the same [1]. In addition, due to the rational traffic organization, the HGRN also has the characteristics of good connectivity and selectivity [2]. HGRNs have been implemented in many urban centers around the world including Manhattan (New York City), Barcelona in Spain, Ginza (Tokyo) in Japan as well as the Bund area in Shanghai, the Xinjiekou area in Nanjing, and others.

Recently, regional traffic signal synchronization has become one of the main research directions in the field of urban traffic signal control, and some regional traffic signal control systems have been developed, such as TRANSYT, SCATS, and SCOOT. Unfortunately, when applied in the saturated HGRN, the performance of these systems has not been satisfactory. When the network is saturated, there is no extra time and space to optimize the traffic signals. Therefore, the regional signal control systems cannot optimize the signal control parameters at the intersections, and the control systems may operate as fixed-timed control systems. In this situation, the traffic system is more fragile and prone to traffic congestion.

Besides, the signalized intersections are densely distributed, and the accommodation space for the vehicle queues is limited. As a result, if congestion occurs at one intersection, the congestion will cause a domino effect, which may cause the regional congestion in the HGRN. Meanwhile, once it happens, the mobility in the HGRN will be difficult to restore.

This research aims at proposing a regional traffic signal synchronization strategy for the saturated HGRN to alleviate traffic congestion and to improve traffic efficiency in the saturated network. The paper is organized as follows.  Section 2 summarizes research results in the field of the regional traffic signal synchronization. Then, the long green and long red (LGLR) traffic control strategy for the saturated HGRN is proposed and analyzed in Section 3.  Section 4 presents the LGLR traffic signal synchronization model. In Section 5, the application of the control model is simulated in the saturated HGRN and the performance of the control model is analyzed. Finally, the advantages and disadvantages of the LGLR traffic control strategy are discussed and further studies are proposed in Section 6.

## 2. Literature Review

In the past several decades, a variety of deterministic and/or stochastic models have been developed to solve complex traffic and transportation engineering problems. Some traffic signal synchronization strategies have been applied practically, and others are still in the research stage. In this section, various models of traffic signal synchronization are reviewed.

It is by now well established that traffic signal synchronization is an effective measure for reducing traffic congestion; hence a great effort has been made in the area of signal timing optimization techniques. Most of these control strategies are based on fixed-time signal control, including Webster's model [3], semigraphical model [4], Pontryagin's control model [5], and store and forward model [6]. However, fixed-time signal control strategies are only applicable to undersaturated traffic conditions, whereby vehicle queues are only generated during the red phases and are dissolved during the green phases. The main drawback of fixed-time strategies is that their settings are based on historical data rather than real-time data. This may be a crude simplification because demands may vary on different days due to special events.

Regional coordinated traffic control strategies can synchronize traffic signals at the coordinated intersections to improve system performance. Regional coordinated traffic control strategies have been proposed by many researchers, which mainly employ artificial intelligence algorithms, including genetic algorithms, fuzzy logic algorithms, neural network algorithms, and mixed-integer linear programming.

Yu and Recker proposed an adaptive control model of a network of signalized intersections based on a discrete-time, stationary, Markov decision process. The model incorporated probabilistic forecasts of individual vehicle actuations at downstream inductance loop detectors. However, in order to be directly applicable, this proposed model requires complete information on the transition probabilities of the system, which is often not available [7].

Akiyama and Okushima modified the inflow traffic controller with continuous variables to optimize parameters for linguistic expression in fuzzy reasoning and proposed an advanced fuzzy traffic control as an extension of conventional inflow control traffic management to reduce the traffic congestion effectively on urban expressways in Japan [8].

Srinivasan et al. adopted the multiagent system approach to develop distributed unsupervised traffic responsive signal control models, where each agent in the system is a local traffic signal synchronizer for one intersection in the traffic network. The first multiagent system is developed using hybrid computational intelligent techniques. The second multiagent system is developed by integrating the simultaneous perturbation stochastic approximation theorem in fuzzy neural networks [9].

Li et al. presented a new signal control method based on a model-free action-dependent adaptive dynamic programming. This method could be used for cooperative control of multiple intersections. In each intersection, the signal controller was adopted to adjust signal time according to an integrated unity parameter. The unity parameter was designed to consider not only the control performance in local intersection but also those in the neighbor intersections [10].

Gokulan and Srinivasan proposed a distributed multiagent-based approach to develop a traffic-responsive signal control system, that is, the geometric fuzzy multiagent system. This system was capable of handling the various levels of uncertainty found in the inputs and rule base of the traffic signal synchronizer. Simulation models of the agents designed in PARAMICS were tested on virtual road network replicating a section of the central business district in Singapore [11].

Sánchez-Medina et al. developed and tested a new model for traffic signal optimization based on the combination of three key techniques: genetic algorithms for the optimization task; cellular-automata-based microsimulators for evaluating each possible solution for traffic-light programming times; and a Beowulf Cluster, which was a multiple-instruction-multiple-data multicomputer of excellent price/performance ratio [12].

Chen and Khorasani developed a robust decentralized congestion control strategy for a large scale network with differentiated services traffic. The proposed congestion controller did take into account the associated physical network resource limitations and was shown to be robust to the unknown and time-varying delays. This strategy was developed on the basis of differentiated services architecture by utilizing a robust adaptive technique. A linear matrix inequality condition was obtained to guarantee the ultimate boundedness of the closed-loop system [13].

Yun and Park presented a stochastic-optimization method for coordinated actuated traffic signal systems. The proposed method accounts for stochastic variability by using a well-calibrated microscopic simulation model, CORSIM, instead of a macroscopic and deterministic model, and it simultaneously optimizes actuated signal settings and the four traffic signal timing parameters by adopting a genetic algorithm with special decoding schemes. The proposed method has been applied to a real-world arterial network in Charlottescille, Virginia. The results indicated that the proposed method outperforms the existing timing plan and synchro-optimized traffic signal timing for the tested arterial network [14].

Varaiya introduced the max pressure (MP) control. At each intersection, MP selects a stage that depends only on the queues adjacent to the intersection. MP does require knowledge of mean turn ratios and saturation rates, but an adaptive version of MP will have the same performance, if turn movements and saturation rates can be measured. The advantage of MP over other SF network control formulations is that it only requires local information at each intersection and provably maximizes throughput [15].

Li proved that there exist infinite optimal solutions in the MAXBAND model if a known optimal solution holds some properties. Li developed a two-phase approach: in the first phase, he solved the MAXBAND models with perturbation controlled by a parameter and generated a number of optimal or suboptimal plans, and in the second phase, he applied the Monte Carlo method to simulate random progression time, evaluate the generated plans, and rank them by the reliability [16].

In addition to those theoretical models, there are some coordinated traffic control strategies that have been applied practically. MAXBAND [17] is a bandwidth optimization program for arterials and triangular networks. TRANSYT-7F [18] is developed to optimize the signal control parameters for urban road networks. SYNCHRO [19] is a macroscopic analysis and optimization program based on ICU 2003 and HCM 2000. SCOOT [20] and SCATS [21] are two well-known and widely used coordinated traffic responsive strategies.

However, most of the existing algorithms for signal coordination do not explicitly consider saturated situations, because most of research has been devoted to the development of signal control algorithms under normal traffic conditions. Practical procedures or guidelines for signal timing of saturated network are not readily available [22]. As a result, implementation of the algorithms for saturated networks has caused undesirable outcomes. SCOOT, for example, has performed well in moderate traffic conditions but has shown major deficiencies in saturated and highly fluctuating conditions [23]. In addition, it is proved that SCATS is more effective at reducing delay during low volume periods than high volume periods.

## 3. Control Strategy

The long green and long red (LGLR) traffic signal synchronization strategy for the saturated HGRN is proposed in this section. In order to quickly remove queues and to improve traffic efficiency and stability, the approach of the control strategy is to control the formation and dissipation of queues and to maintain the continuous traffic flow.

### 3.1. The LGLR Traffic Signal Synchronization Strategy

The LGLR traffic signal synchronization strategy uses the same signal control timing plan at all signalized intersections in the HGRN, and the straight phases of the control timing plan have a long green time and a long red time. There are two statuses for horizontal direction and vertical direction of the HGRN in this strategy. More specifically, status one is long green (LG). In LG status, the straight phases in horizontal direction of all signalized intersections in the HGRN are all green, and green lights last for a long time. The straight vehicles in horizontal direction can form continuous traffic flows, maintain stable travel speeds, and go uninterrupted through several signalized intersections. Status two is long red (LR). In LR status, the straight phases in horizontal direction all change from green to red and red lights last for a long time. The straight vehicles in horizontal direction can stop at the stop lines at different intersections to avoid congestion when there are too many vehicles from the upstream roads. The statuses in vertical direction are similar to the statuses in horizontal direction.

### 3.2. Application Feasibility

This feasibility analysis assumes that the LGLR traffic signal synchronization strategy is used in a horizontal road including four adjacent signalized intersections, the lengths of sections between adjacent intersections are all short, and there are no turning vehicles entering and exiting the horizontal road. To simplify the analysis process, two-phase traffic signal synchronization is studied.

When the horizontal straight phases of all intersections are LR status, horizontal vehicles stop at the stop lines, and the number of stopping vehicles in each section is stable. Then the horizontal straight phases of all intersections change from LR status to LG status and the horizontal vehicles begin to travel. When the horizontal straight phases are LG status, the traffic flow will not be interrupted by the intersections, so the road can be regarded as a road without intersections. At first, the traffic flow on the road is discrete, but after a short time, the flow will form a continuous traffic flow.

Because the traffic flow is continuous when the horizontal straight phases of all intersections are LG status, the Greenshields et al.'s linear speed-density model can be used [24] to describe the average traffic density of the traffic flow on the road and formula (1) is derived:

(1) $$\begin{array}{c} K=K_{j}\left(1-\frac{V}{V_{f}}\right), \end{array}$$

where *K* is the average density of the flow on the road, *K*_*j*_ is the congestion density on the road, *V* is the average travel speed of the continuous traffic flow on the road, and *V*_*f*_ is the free flow speed on the road.

According to formula (1), the average number of vehicles on each section is described as

(2) $$\begin{array}{c} N_{i}=L_{i}K=L_{i}K_{j}\left(1-\frac{V}{V_{f}}\right), \end{array}$$

where *N*_*i*_ is the average number of vehicles on the section *i* and *L*_*i*_ is the length of the section *i*.

When the horizontal straight phases of all intersections change from LG status to LR status, vehicles on each section stop at the stop line and form a queue. The queue length on each section is described as

(3) $$\begin{array}{c} q_{i}={\overset{-}{h}}_{s}N_{i}={\overset{-}{h}}_{s}L_{i}K_{j}\left(1-\frac{V}{V_{f}}\right), \end{array}$$

where *q*_*i*_ is the queue length on the section *i* and ${\overset{-}{h}}_{s}$ is the average space headway of the queue.

Considering the relationship of the average space headway of the queue and the congestion density, the average space headway of the queue can be obtained by

(4) $$\begin{array}{c} {\overset{-}{h}}_{s}=\frac{1}{K_{j}}. \end{array}$$

Substitute formula (4) into formula (1):

(5) $$\begin{array}{c} q_{i}=L_{i}\left(1-\frac{V}{V_{f}}\right). \end{array}$$

When the horizontal straight phases are LG status, it is assumed that vehicles travel at the same speed *V* on the road. After the horizontal straight phases of all intersections change from LR status to LG status, the time, when the head of the queue on the upstream section approaches the end of the queue on the downstream section, is determined as

(6) $$\begin{array}{c} T_{i}^{\prime }=\frac{L_{i}-q_{i}}{V}=\frac{L_{i}}{V_{f}}. \end{array}$$

The time when the end of the queue on the downstream section begins to move is determined as

(7) $$\begin{array}{c} T_{i}^{\prime \prime }=\frac{q_{i}}{u}, \end{array}$$

where *u* is the starting wave speed of the queue.

According to traffic flow theory, the starting wave speed is determined as

(8) $$\begin{array}{c} u=V_{f}-V. \end{array}$$

Substitute formulas (5) and (8) into formula (7):

(9) $$\begin{array}{c} T_{i}^{\prime \prime }=\frac{L_{i}}{V_{f}}. \end{array}$$

According to formulas (6) and (9), the following is obtained:

(10) $$\begin{array}{c} \Delta T_{i}=T_{i}^{\prime }-T_{i}^{\prime \prime }=0, \end{array}$$

where Δ*T*_*i*_ is the lost time of upstream and downstream queues connecting to each other.

Δ*T*_*i*_ > 0 means that the end of the downstream queue begins to move before the head of the upstream queue approaches the position at the end of the downstream queue and Δ*T*_*i*_ < 0 means that the end of the downstream queue does not move when the head of the upstream queue approaches the position at the end of the downstream queue. It is concluded that there is a lost time between upstream and downstream queues when Δ*T*_*i*_ > 0 or Δ*T*_*i*_ < 0.

Δ*T*_*i*_ = 0 means that the end of the downstream queue begins to move, while the head of the upstream queue approaches the position of the end of the downstream queue. The upstream and downstream queues seamlessly connect to each other and restore a continuous traffic flow without any lost time.

**Figure 1:**
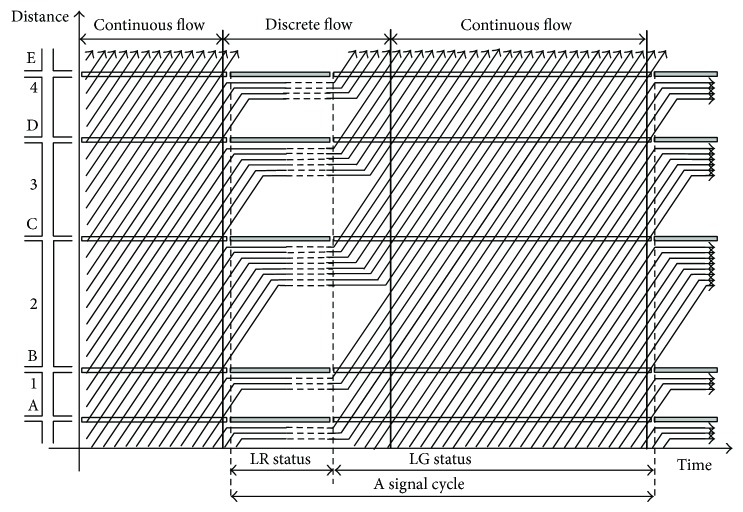
The LGLR traffic signal synchronization strategy in use on a road.

Therefore, according to formula (10), it is concluded that, after the horizontal straight phases of all intersections change from LR status to LG status, if the length of the green time is longer than $\underset{}{\max}T_{i}^{\prime }$, the queues can become a continuous traffic flow at $\underset{}{\max}T_{i}^{\prime }$. In addition, the lengths of sections are all short, which ensures that the traffic flow is continuous during most of the duration of LG status (see Figure 1).

As shown in Figure 1, when the straight phases of all intersections change from LG status to LR status, the continuous traffic flow will be interrupted at the signalized intersections, and the queues are formed at the stop lines on the sections. The lengths of queues are unchanged during the LR status. When the straight phases of all intersections change from LR status to LG status, the discrete traffic flow on the road becomes a continuous traffic flow after *T*_2_′, because the length of section 2 is longest. According to formula (10), during the process of forming the continuous traffic flow, there is no lost time. When the traffic flow is continuous, vehicles can pass through several signalized intersections without stopping.

If the area controlled by the LGLR traffic signal synchronization strategy extends to the HGRN, the strategy will also work effectively. When the strategy is used in the HGRN, the same signal control timing plan will be applied in all signalized intersections. In a signal cycle, when the horizontal straight phases in the HGRN are LG status (i.e., the vertical straight phases are LR status), the horizontal straight vehicles form continuous traffic flows and pass through several downstream signalized intersections at the steady travel speed. Meanwhile, the vertical straight vehicles stop at each section and wait for the green light. When the vertical straight phases in the HGRN change to LG status (i.e., the horizontal straight phases are LR status), the vertical straight vehicles form continuous traffic flows to travel, and the horizontal straight vehicles stop and wait for the green light. Additionally, the same signal control timing plan is applied to all signalized intersections of the HGRN; therefore, the computational burden can be reduced and the optimal signal control timing plan can be easily generated at the control center.

Therefore, it is concluded that the LGLR traffic signal synchronization strategy is feasible in the HGRN.

## 4. Modeling

Traffic organization in the HGRN needs to be considered with the application of the LGLR traffic signal synchronization strategy. At present, the four-phase signal control is commonly used at the signalized intersections in the HGRN in China, and the four-phase signal control is one of the widely used traffic signal synchronizations in the world. Therefore, based on the four-phase signal control, the LGLR traffic signal synchronization model is proposed.

The purpose of using the LGLR traffic signal synchronization strategy in the HGRN is to maintain continuous traffic flows when vehicles travel and avoid excessive queuing when vehicles stop and wait for the green light. The objective of the model is to minimize the number of stops, because the number of stops not only reflects the continuity of the traffic flow, but also closely relates to traffic capacity, rear-end accidents, fuel consumption, exhaust emissions, noise pollution, and other congestion issues.

### 4.1. Model Objective Function

When the HGRN is saturated, the distribution of saturation flow rates on all parallel roads is uniform in the HGRN, and the traffic flows of all roads are stable without significant fluctuations [25]. According to the process of LGLR traffic signal synchronization strategy (formulas (1)~(10)), when the traffic flow on the lane travels through this area, the number of stops for a lane only relates to the road length, the average speed of the continuous traffic flow, the time interval of LG status, and the traffic volume. In addition, the number of intersections and the distances between adjacent intersections will not influence the number of stops. The number of stops in the west-to-east lane *i* (Stop_WE_^*i*^) is described as

(11) $$\begin{array}{c} \text{Sto}{\text{p}}_{\text{WE}}^{i}=\frac{L_{\text{ho}}}{V_{\text{WE}}^{}G_{\text{ho}}}\cdot Q_{\text{WE}}^{i}, \end{array}$$

where *L*_ho_ is the average length of the horizontal roads in the HGRN, *G*_ho_ is the time interval of LG status for the horizontal straight phases in the HGRN, *Q*_WE_^*i*^ is the traffic volume in the west-to-east lane *i*, and *V*_WE_ is the average speed of the continuous traffic flow when the horizontal straight phases are green.

According to formula (11), the number of stops from west to east (Stop_WE_) is described as

(12) $$\begin{array}{c} \text{Sto}{\text{p}}_{\text{WE}}=\underset{i}{\sum }\text{Sto}{\text{p}}_{\text{WE}}^{i}=\frac{L_{\text{ho}}}{V_{\text{WE}}^{}G_{\text{ho}}}\cdot \underset{i}{\sum }Q_{\text{WE}}^{i}=\frac{L_{\text{ho}}}{V_{\text{WE}}^{}G_{\text{ho}}}\cdot Q_{\text{WE}}^{}, \end{array}$$

where *Q*_WE_ is the total traffic volume from west to east.

The average number of vehicle stops on the horizontal and vertical roads in the HGRN (${\overset{-}{N}}_{\text{stop}}^{\prime }$) is described as

(13) N−stop′=LhoVWEGhoQWE+LhoVEWGhoQEWpppp+LveVNSGveQNS+LveVSNGveQSN×Q−1,

where *G*_ve_ is the time interval of LG status for the vertical straight phases in the HGRN, *L*_ve_ is the average length of the vertical roads in the HGRN, *Q*_EW_, *Q*_NS_, and *Q*_SN_ are the total traffic volumes from east to west, from north to south, and from south to north, respectively, and *Q* is the total traffic volume in the HGRN, *Q* = *Q*_WE_ + *Q*_EW_ + *Q*_NS_ + *Q*_SN_.

Considering the relationship of the speed, density, and volume of the continuous traffic flow, formula (13) is simplified as follows:

(14) N−stop′=NWE+NEW/Gho+NNS+NSN/GveQ=Nho/Gho+Nve/GveQ,

where *N*_WE_, *N*_EW_, *N*_NS_, and *N*_SN_ are the numbers of vehicles on the roads from west to east, from east to west, from north to south, and from south to north in the HGRN, respectively, and *N*_ho_ and *N*_ve_ are the numbers of vehicles on the horizontal and vertical roads, respectively, *N*_ho_ = *N*_WE_ + *N*_EW_, *N*_ve_ = *N*_NS_ + *N*_SN_.

However, formula (13) does not include all stops in the HGRN, because there are left-turn vehicles in the HGRN. When the straight phases are LG status, these vehicles go straight until they approach the intersections where the vehicles need to stop and wait for the green left-turn signals. According to formula (10), after the left-turn signals turn green, the left-turn vehicles will approach the end of the queues and stop again at the next intersections.

In addition, with the increase of the density of the HGRN, the average left-turn ratio decreases and the distribution of left-turn vehicles is more uniform [25]. Therefore, compared with formula (14), if the green time, during a signal cycle, for the left-turn phase is long enough for left-turn vehicles, each left-turn vehicle will add a new stop, and the average number of new stops of left-turn vehicles (*Ω*) is described as

(15) $$\begin{array}{c} \Omega =\frac{\eta_{\text{W}}^{L}Q_{\text{WE}}+\eta_{\text{E}}^{L}Q_{\text{EW}}+\eta_{\text{N}}^{L}Q_{\text{NS}}+\eta_{\text{S}}^{L}Q_{\text{SN}}}{Q}, \end{array}$$

where *η*_W_^*L*^, *η*_E_^*L*^, *η*_N_^*L*^, and *η*_S_^*L*^ are the left-turn ratios in the west, the east, the north, and the south at intersections, respectively.

According to formulas (14) and (15), the objective function of the average number of stops in the HGRN (${\overset{-}{N}}_{\text{stop}}$) is shown as

(16) N−stop=min⁡N−stop′+Ω=min⁡Nho/Gho+Nve/GveQ+Ω.

### 4.2. Constraints

In the model, the constraints include no more than one stop for each left-turn vehicle, the accommodation space for vehicles around the HGRN, the waiting tolerance of participants, and the stability of continuous traffic flows.

#### 4.2.1. The Constraint of No More Than One Stop for Each Left-Turn Vehicle

In order to balance the traffic flows in different directions, it is necessary to ensure that most left-turn vehicles stop no more than once to turn left at the signalized intersections. In addition, formula (15) is correct only when the green time for the left-turn phase is long enough for left-turn vehicles during a signal cycle. Therefore, the LGLR traffic signal synchronization model must satisfy

(17) $$\begin{array}{c} G_{\text{ho}}^{L}=\max\left(\frac{\eta_{\text{W}}^{L}Q_{\text{WE}}G_{\text{ho}}}{3600n}\cdot {\overset{-}{h}}^{L},\frac{\eta_{\text{E}}^{L}Q_{\text{EW}}G_{\text{ho}}}{3600n}\cdot {\overset{-}{h}}^{L},{\underline{T}}^{L}\right),\\ G_{\text{ve}}^{L}=\max\left(\frac{\eta_{\text{N}}^{L}Q_{\text{NS}}G_{\text{ve}}}{3600n}\cdot {\overset{-}{h}}^{L},\frac{\eta_{\text{S}}^{L}Q_{\text{SN}}G_{\text{ve}}}{3600n}\cdot {\overset{-}{h}}^{L},{\underline{T}}^{L}\right), \end{array}$$

where *G*_ho_^*L*^ and *G*_ve_^*L*^ are the green times for the left-turn phases on the horizontal and vertical roads, respectively, *n* is the number of the signalized intersections in the HGRN, ${\overset{-}{h}}^{L}$ is the average time headway of left-turn vehicles, and ${\underline{T}}^{L}$ is the minimum green time for the left-turn phase.

#### 4.2.2. The Constraint of the Accommodation Space for Vehicles around the HGRN

The signal control timing plan in the HGRN is quite different from that around the HGRN. Within a signal cycle in the HGRN, many vehicles need to leave and enter the HGRN, and these vehicles need enough space to be accommodated in a short time around the HGRN and may cause long queues. Therefore, the constraint of the accommodation space for vehicles around the HGRN is proposed to limit the length of green time for the straight phases, as shown in

(18) $$\begin{array}{c} \frac{G_{\text{ho}}Q_{\text{WE}}}{3600K_{j}}<\underset{i}{\min}\;L_{\text{E}}^{i},\\ \frac{G_{\text{ho}}Q_{\text{EW}}}{3600K_{j}}<\underset{i}{\min}\;L_{\text{W}}^{i},\\ \frac{G_{\text{ve}}Q_{\text{NS}}}{3600K_{j}}<\underset{i}{\min}\;L_{\text{S}}^{i},\\ \frac{G_{\text{ve}}Q_{\text{SN}}}{3600K_{j}}<\underset{i}{\min}\;L_{\text{N}}^{i}, \end{array}$$

where *L*_E_^*i*^, *L*_W_^*i*^, *L*_S_^*i*^, and *L*_N_^*i*^ are the lengths of the roads that connect to the HGRN in four directions, respectively.

#### 4.2.3. The Constraint of the Waiting Tolerance of Participants

When the straight phases on the roads are LR status, participants need to wait for the green light at the intersections. The influences of individual psychology, traffic means, and waiting environment are different, so is the waiting tolerance of different participants. According to the statistics of the United Kingdom and Japan, it is proposed that the average of maximum waiting tolerances should be 150 sec. Therefore, the constraint of the waiting tolerance of participants is shown as

(19) $$\begin{array}{c} C-G_{\text{ho}}\leq 150,\\ C-G_{\text{ve}}\leq 150,\\ C-G_{\text{ho}}^{L}\leq 150,\\ C-G_{\text{ve}}^{L}\leq 150, \end{array}$$

where *C* is the signal cycle time.

#### 4.2.4. The Constraint of the Stability of Continuous Traffic Flows

Given the advantages of the continuous traffic flow, the green time for the straight phases should be long enough to avoid frequent switching of the signals to interrupt the continuous traffic flow at the intersections. In addition, it is proposed that the traffic capacity on the road can be increased by 80% when the distance between the intersections increases from 200 to 800 meters [26], which means the traffic capacity increases with the increase of the distance between two intersections. Therefore, the constraint of the stability of continuous traffic flows is shown as

(20) $$\begin{array}{c} G_{\text{ho}}\min\left(V_{\text{WE}},V_{\text{EW}}\right)\geq \min\left(L_{\min},L_{\text{ho}}\right),\\ G_{\text{ve}}\min\left(V_{\text{NS}},V_{\text{SN}}\right)\geq \min\left(L_{\min},L_{\text{ve}}\right), \end{array}$$

where *L*_min⁡_ is the minimum travel distance of the continuous traffic flow.

Therefore, the LGLR traffic signal synchronization model is constituted with the objective function formula (16) and constraint formulas (17)–(20).

## 5. Simulation

The purpose of the simulation is to analyze the performance of the LGLR traffic signal synchronization strategy used in the saturated HGRN. First, the real-life conditions of the HGRN in Nanjing are considered, and the saturated HGRN is simulated in Vissim. Second, short-time traffic data are collected in Vissim and the solutions of the LGLR traffic signal synchronization model are optimized by Matlab's optimization toolbox to control the signal lights in the HGRN. Finally, the performance of the LGLR traffic signal synchronization model is compared with those of the two other models, and the feasibility of the LGLR traffic signal synchronization strategy is analyzed.

**Figure 2:**
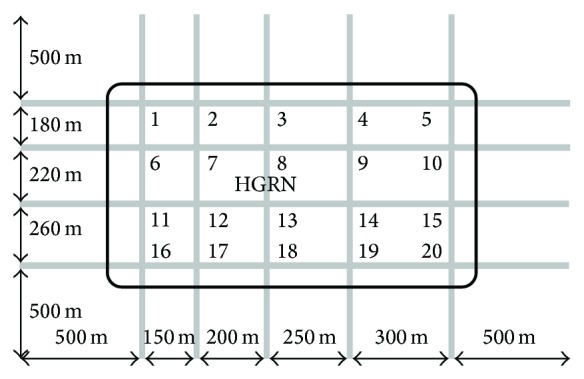
The simulated HGRN.

As shown in Figure 2, the HGRN of Nanjing is simulated in Vissim. In the HGRN, the spacing of the road grid is between 150 and 300 meters, the roads are all four-lane two-way, the approaches are expanded to three lanes, and there are no signification differences in the road grade. Within 20 signalized intersections, four-phase signal control is used, and traffic detectors are installed at the stop lines at signalized intersections. According to the statistics, the average turn-left ratio at the intersections is about 15%, and the saturation flow rate for a lane is 1800 veh/h.

During the simulation process, the signal control parameters at intersections are optimized by the LGLR traffic signal synchronization model according to short-time traffic data, which are collected by traffic detectors in Vissim, and the optimized traffic signal synchronization parameters are used to control the signal lights in the HGRN. The main parameters of the model are set as follows: the average length of the horizontal roads is 900 meters, the average length of the vertical roads is 660 meters, the average time headway of left-turn vehicles is 4 seconds, the minimum green time for the left-turn phase is 8 seconds, the minimum length of each road connecting with the HGRN from four directions is 500 meters, the minimum travel distance of the continuous traffic flow is 600 meters, and the congestion density is 140 veh/km.

An algorithm is developed to analyze the performance of the LGLR traffic signal synchronization model, with the following specific steps.

*Step 1*. Simulate traffic volume of each entrance lane into the HGRN to control the saturation rate of the HGRN. The traffic volume of each entrance lane is set to 1200 veh/h and increases by 200 veh/h each hour. The duration of the simulation is 3 hours. 

*Step 2*. Using the LGLR traffic signal synchronization model, collect traffic data in the HGRN, such as traffic volume, travel speed, and left-turn ratio, and optimize and update the parameters of the signal lights every 15 minutes. 

**Figure 3:**
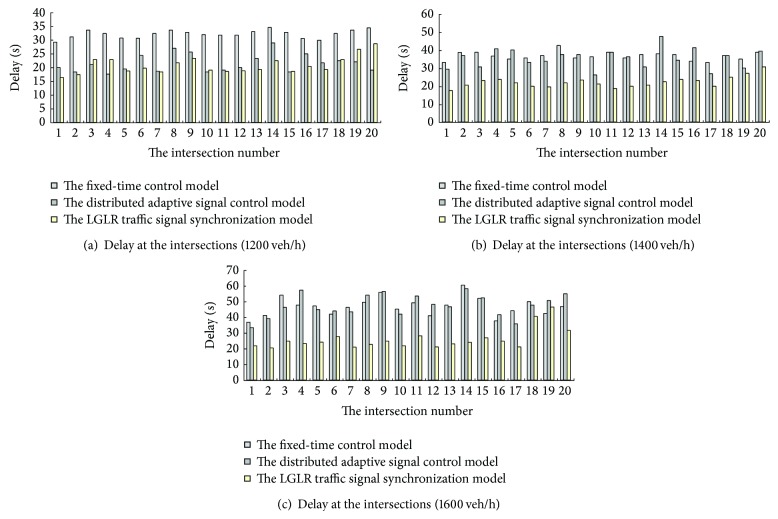
Comparison of delay at the intersections.

**Figure 4:**
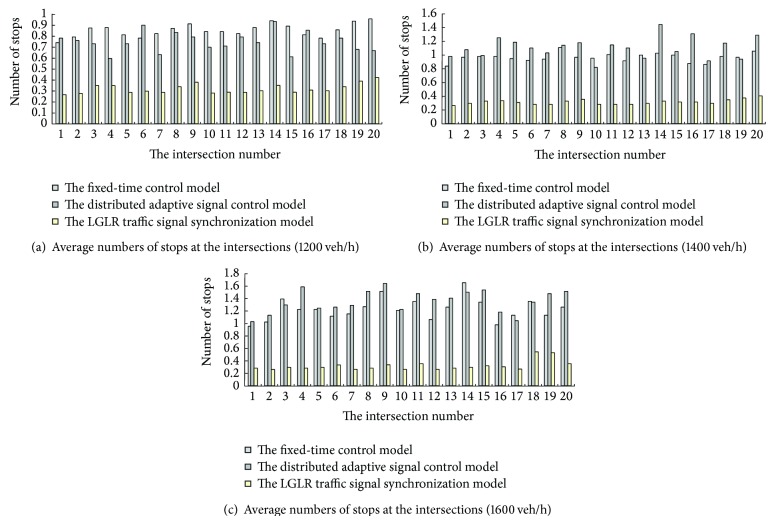
Comparison of average numbers of stops at the intersections.

**Figure 5:**
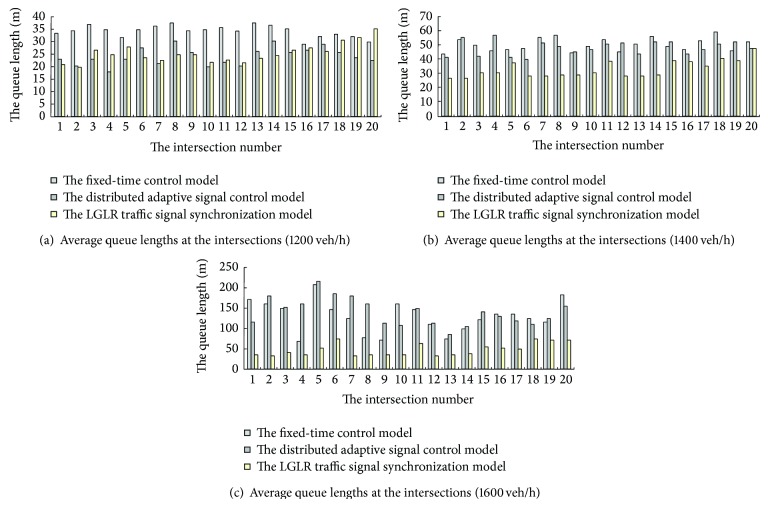
Comparison of average queue lengths at the intersections.

*Step 3*. Compare the performance of the LGLR traffic signal synchronization model used in the saturated HGRN with those of the fixed-time control model and the distributed adaptive signal control model [28], and then analyze the feasibility and advantages of the LGLR traffic signal synchronization strategy (see Figures 3–5).

When the traffic volume of each entrance lane is 1200 veh/h, that is, the HGRN is close to saturation, the performance of the LGLR traffic signal synchronization model and that of the distributed adaptive signal control model are better than the performance of the fixed-time control model. However, the performance of the LGLR traffic signal synchronization model and that of the distributed adaptive signal control model are similar (see Figure 3(a)).

When the traffic volume increases to 1400 veh/h and 1600 veh/h, that is, the HGRN is saturated, the delays of the intersections all increase in the three control models, but the increase of the delays of the intersections in the LGLR traffic signal synchronization model is smallest in three control models (see Figures 3(b) and 3(c)). Meanwhile, when the traffic volumes are 1600 veh/h, the delays in the distributed adaptive signal control model are similar to the delays in the fixed-time control model, which means the distributed adaptive signal control becomes the fixed-timed control in the saturated HGRN.

In terms of the number of stops in the intersections, the superiority of the LGLR traffic signal synchronization model is obvious. In three cases of 1200 veh/h, 1400 veh/h, and 1600 veh/h (see Figures 4(a), 4(b), and 4(c)), the average numbers of stops at all intersections are less than 0.6 in the LGLR model and are obviously less than those in other models.

Additionally, in the cases of 1400 veh/h and 1600 veh/h (Figures 4(b) and 4(c)), the numbers of stops in the distributed adaptive signal control model and the fixed-time control model are not less than one, which means almost every vehicle must stop at least once at each intersection.

In terms of average queue lengths at the intersections, because the optimization objective of the distributed adaptive signal control model is the minimum average queue lengths, the performance of the distributed adaptive signal control model is slightly better than that of other models in the case of 1200 veh/h (Figure 5(a)). However, when the traffic volumes increase, the performance of the LGLR traffic signal synchronization model gets better, which means the probability of spillback congestion is smaller. Meanwhile the performance of the other two models remains similar under all traffic volumes, and average queue lengths at the intersections are not controlled (see Figures 5(b) and 5(c)).

As shown in Table 1, all three models are evaluated in terms of their overall performance in the saturated HGRN. In the evaluations, total number of vehicles, total travel distance, average travel time, average travel speed, and average delay are included. In the case of 1200 veh/h, it is difficult to show the superiority of the LGLR traffic signal synchronization model, because the performance of the LGLR traffic signal synchronization model at this volume is worse than other models. However, in the cases of 1400 veh/h and 1600 veh/h (when the HGRNs are saturated), the superiority of the LGLR traffic signal synchronization model is gradually revealed, and the performance is obviously better than those of other controls. For example, compared with the performance of the distributed adaptive signal control model, with the slight increase of the total number of vehicles and the total travel distance, the average travel time decreases by 25.5%, average travel speed increases by 33.3%, and average delay decreases by 39.6%.

## 6. Conclusions

The time and space for traffic signal optimization are limited in the saturated HGRN, so the performance of conventional signal control methods is not satisfactory. Therefore, the LGLR traffic signal synchronization strategy is proposed as an alternative. This strategy uses the same signal control timing plan to control all signalized intersections. The green time and the red time for the straight phases of the timing plan are relatively long to limit the queue lengths at all intersections when the lights are red and to ensure that vehicles can form the continuous traffic flow and go through several downstream intersections without stopping when the lights are green. The performances of three signal control models were compared and analyzed by simulations, and results showed that, in the saturated HGRN, the LGLR traffic signal synchronization strategy is much more effective, for the following reasons.

- First, in the LGLR traffic signal synchronization strategy, the same signal control timing plan is used at all intersections, which is helpful to uniformly distribute the traffic volumes in the HGRN and to fulfill the advantages of good equilibrium, connectivity, and selectivity of the HGRN.
- Second, when the straight phases of the intersections are LR status, the straight vehicles stop at the stop lines at different intersections in order to limit the queue lengths in the sections and to avoid the spillback congestions to the upstream intersections.
- Third, when the straight phases of the intersections are LG status, the straight vehicles can form continuous traffic flows and go uninterruptedly through several downstream signalized intersections at a steady speed.
- Finally, the optimization is simple, as the traffic parameters of the model can be obtained by the traffic detectors installed in the HGRN, and this requirement of the hardware and software is easy to implement.

In short, the essence of the LGLR traffic signal synchronization strategy is to control the formation and dissipation of queues and to maximize the efficiency of traffic flow at signalized intersections in the saturated HGRN, which is the same as Roess et al.'s point that the formation of queues and blockages is inevitable during saturation and removal of queues and blockages must be the prime objectives [29].

Moreover, according to Section 3.2, the LGLR traffic signal synchronization strategy is applicable not only in the HGRN, but also in the corridor or parallel corridors. If weight coefficients are introduced into the modeling of the strategy to focus on saturated traffic flow in the direction of corridors, the strategy may be adopted in the corridor or parallel corridors.

However, the LGLR traffic signal synchronization strategy is not suitable to the undersaturated HGRN, because, in this scenario, the LGLR traffic signal synchronization strategy cannot use the extra time and space to optimize the control signal parameters. As shown in Table 1, when the network is close to saturation (in the case of 1200 veh/h), the control effort of this strategy is slightly worse than others until the network reaches saturation (as shown in the cases of 1400 veh/h and 1600 veh/h).

To expand the application and widespread use of the LGLR traffic signal synchronization strategy, some details of the strategy require further research.

- First, the coordinated traffic signal synchronization in the area around the HGRN needs to be studied to ensure the stability of traffic between the HGRN and its surrounding road network.
- Second, the average of maximum waiting tolerance time for different participants using different modes, such as driver, bicyclist, and pedestrian, needs to be studied.

## Figures and Tables

**Table 1:** Overall evaluation of the application of three control models in the HGRN [27].

Evaluation indexes	Signal control methods	1200 veh/h	1400 veh/h	1600 veh/h
Total number of vehicles (veh)	Fixed-time signal control	19956	23054	24517
	Distributed adaptive signal control	20247	22718	25443
	LGLR signal control	20256	23507	26604

Total travel distance (km)	Fixed-time signal control	34046.03	39290.68	41697.06
	Distributed adaptive signal control	34566.26	38660.45	43258.30
	LGLR signal control	34586.78	40115.42	45379.62

Average travel time (sec)	Fixed-time signal control	255.58	279.29	375.69
	Distributed adaptive signal control	221.49	295.08	368.92
	LGLR signal control	226.68	235.45	259.37

Average travel speed (km/h)	Fixed-time signal control	24.03	21.97	16.29
	Distributed adaptive signal control	27.74	20.76	16.59
	LGLR signal control	27.12	26.10	23.68

Average delay (sec)	Fixed-time signal control	138.52	162.35	259.18
	Distributed adaptive signal control	104.37	178.39	252.46
	LGLR signal control	109.37	118.10	142.02
